# Proliferative epithelial changes in tumour adjacent tissue in Sri Lankan women with breast carcinoma: do morphological changes support molecular models of breast carcinogenesis?

**DOI:** 10.1186/s13000-022-01281-w

**Published:** 2022-12-29

**Authors:** Indumini Maheshika Jinadasa, Harshima Disvini Wijesinghe, Modini Manohari Abeydera Jayawickrama, Menaka Dilani Samarawickrama Lokuhetty

**Affiliations:** 1 Department of Histopathology, National Hospital of Sri Lanka, Colombo, Sri Lanka; 2 Department of Pathology, Faculty of Medicine, University of Colombo, Colombo, Sri Lanka

**Keywords:** Breast carcinoma, Columnar cell lesions, DCIS, Flat epithelial atypia, ER

## Abstract

**Background:**

The multistep molecular model of breast carcinogenesis is based on the oestrogen receptor(ER) status of the tumour. Its two main arms comprise ER-positive and ER-negative breast carcinomas(BCa), which are associated with Nottingham grade(NG) of the tumour and different proliferative epithelial changes. According to the model, columnar cell lesions(CCL), lobular carcinoma in-situ(LCIS) and atypical ductal hyperplasia(ADH), low-grade ductal carcinoma in-situ (LG-DCIS) are associated with low grade ER-positive tumours and microglandular adenosis (MGA), pleomorphic LCIS(PLCIS), high-grade DCIS(HG-DCIS) are associated with ER-negative high grade tumours. This study aims to describe the association between proliferative epithelial changes in breast tissue adjacent to tumour, in relation to the ER status and NG of the tumour.

**Methods:**

This descriptive cross-sectional study included 420, wide local excision and mastectomy specimens of BCa from National Hospital of Sri Lanka, between 2017–2019. The histopathological features of the tumour and proliferative epithelial changes in tumour adjacent tissue within 10 mm distance from the tumour-host interface were evaluated independently by two pathologists. The ER, PR(Progesterone receptor) and HER2 status assessed by immunohistochemistry(IHC) was reviewed. The associations between above epithelial lesions and ER status and NG{categorised as low grade (NG1 and NG2) and high grade (NG3)} of the tumour were analyzed.

**Results:**

ER positive BCa showed significant associations with CCH (*p* = 0.04), FEA (*p* = 0.035) and LGDCIS (*p* < 0.001). Although PLCIS was more frequent in ER positive tumours, the association did not attain statistical significance. ER negative BCa showed a significant association with HGDCIS (*p* = 0.016). CCLs as a whole (*p* = 0.005) and also CCC (*p* = 0.006) and FEA (*p* = 0.048) and LGDCIS (*p* < 0.001) showed significant associations with low NG tumours. High NG tumours showed a significant association with HGDCIS (*p* < 0.001). Microglandular adenosis was not identified in our study population.

**Conclusion:**

These morphological findings support the multistep molecular based pathogenetic pathways of breast carcinoma in the studied setting in South Asia. Identification of these proliferative epithelial components in a core biopsy that is negative for BCa, should prompt for close clinicoradiological correlation, and if necessary re-biopsy of women suspected of harbouring a BCa.

## Background

Breast cancer (BCa) is the most common cancer in females globally and the leading cause of cancer and cancer related deaths of females in developing countries, including Sri Lanka [1–3]. The necessity for optimal therapeutic targets has fueled many molecular and genomic studies to understand the aetiological heterogeneity in the steps of breast carcinogenesis [4–9]. Distinct proliferative epithelial changes of the breast centered in the terminal duct lobular unit are thought to play a role in the development of invasive cancer [4, 5].

Columnar cell lesions (CCL) especially flat epithelial atypia (FEA), atypical lobular hyperplasia (ALH), lobular carcinoma in situ (LCIS), atypical ductal hyperplasia (ADH) and low-grade ductal carcinoma in situ (LG DCIS) are low grade precursor lesions. Microglandular adenosis (MGA), pleomorphic lobular carcinoma in situ (PLCIS) and high-grade ductal carcinoma in situ (HG DCIS) are high grade precursor lesions [4]. Sclerosing adenosis (SA), radial scar and usual ductal hyperplasia (UDH) are not classified as precursor lesions but are risk indicators [4, 5, 8].

**Fig. 1:**
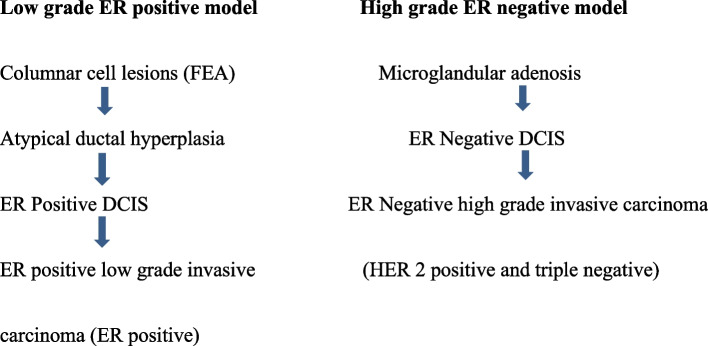
Diagrammatic representation of the two molecular based breast cancer pathogenetic pathways

Research evidence points towards two distinct molecular genetic pathways of breast carcinogenesis that are strongly associated with the tumour grade and oestrogen receptor (ER) status. This novel multistep model of breast carcinogenesis has two main arms comprising ER positive and ER negative tumours [4, 5, 7, 8] (Fig. 1).

The ER positive arm comprises CCL/FEA, ADH/ ER positive DCIS and ALH/LCIS that progress towards the luminal type, ER positive, low grade invasive breast carcinoma (BCa). These ER positive BCa’s of the low-grade breast neoplasia family express hormone receptors, lack HER2 overexpression and are negative for basal markers. Diploid/near diploid simple karyotypes predominate within this group and more than 80% of the tumours contain 16q deletions together with gains of 1q (> 75%) and 16p (> 50%). This group of tumours also includes carcinomas that have progressed from low grade to high grade (Grade 3 invasive breast carcinomas – ER positive) [4, 5, 7–9].

The ER negative arm comprises MGA, ER negative DCIS and PLCIS that progress to ER negative high grade BCa (Grade 3 invasive breast carcinomas). These tumours lack hormone receptors, express HER 2 and/or basal markers and show complex aneuploidy karyotypes. They have losses of 1p, 8p and 17p with gains of 1q and 8q. The 16q deletion which is the hallmark of low-grade breast neoplasia family constitutes only 30% of high-grade tumours [4, 5, 7–9].

In different geographical settings, the aetiological factors and precursor lesions for BCa may vary based on genetic/ethnic and environmental differences. A detailed morphological study comparing the association of different precursor lesions and risk indicators in low-grade and high-grade BCa has not been done previously in the Sri Lankan setting. Additionally, most studies carried out worldwide have focused mainly on the low grade breast neoplasia family with fewer studies on high grade tumours.

The objective of the study was to describe the spectrum of proliferative epithelial changes in tissue adjacent to breast carcinoma in a cohort of Sri Lankan women and to determine the associations between these epithelial changes with ER status of the tumour and the Nottingham grade (NG).

## Methodology

### Study design and setting

This was an analytical cross-sectional study conducted at the Department of Pathology, National Hospital of Sri Lanka (NHSL) and University of Colombo. Ethical Clearance was taken from the Ethical Review Committee of the National Hospital of Sri Lanka (Ref AAJ/ETH/COM/2020/JULY).

### Study materials

A consecutive sample of 420 wide local excision and mastectomy specimens for breast carcinoma were collected retrospectively from NHSL from December 2019, excluding tumours fulfilling the exclusion criteria. The sample size calculation was performed using the formula N = Z^2^ p (1-p)/d^2^ resulting in a sample size of 420, when the standard normal deviates for the selected level of confidence was taken as 1.96 for a confidence level of 95% (Z), with the expected proportion taken as 50% in order to get the maximum sample size (P).Required level of precision was taken as 5% allowing for a non-response rate of 10% (d) [10].

Exclusion criteria were inadequate amount of tumour adjacent non-cancer tissue for assessment in the tissue sections. The tumour adjacent tissue was defined as tissue within 10 mm distance (5 medium power fields [× 10 – 2.0 mm field diameter]) from the tumour host margin, Others included post-neoadjuvant therapy specimens, re-excision specimens due to margin positivity, lack of data on hormone receptor and HER2 status and the male sex.

### Assessment of pathological features

The haematoxylin and eosin-stained slides of all cases were reviewed independently by two pathologists for characteristics of the tumour and the epithelial changes in tumour adjacent tissue.

The histological typing of the tumour was based on the World Health Organization (WHO) 5^th^ series [11]. The grading was done according to Modified Scarff, Bloom and Richardson grade (Nottingham grade-NG) [11, 12]. Tumours of Nottingham grades 1 and 2 were categorized as low grade tumours and tumours of Nottingham grade 3 were categorized as high grade tumours for study purposes.

The immunohistochemistry stained ER, PR and HER2 slides were reviewed. Tumours showing nuclear staining in >  = 1% of tumour cells were categorized as positive for ER/PR [12]. The ASCO—CAP guideline of 2018 was used for the interpretation of HER2 [13]. Tumours that were equivocal for HER2 (HER2–2 +) by IHC were excluded since fluorescent in situ hybridization (FISH) results were inaccessible. The tumours were broadly categorized into luminal, HER2 and triple negative BCa (TNBC) groups based on ER, PR and HER2 status. Further classification of the luminal subgroup into luminal A and B was not attempted as the proliferative index by Ki67 was not available in all cases.

The tumour adjacent tissue was evaluated for the presence of usual ductal hyperplasia, sclerosing adenosis, radial scar (risk indicators), columnar cell lesions, LCIS, ADH, LG DCIS (low grade precursor lesions), microglandular adenosis, and pleomorphic LCIS and HG DCIS (high grade precursor lesions).

### Data analysis

Statistical package for social sciences (SPSS) version 17 was used for analysis. The association between ER status and Nottingham grade with each of the risk indicators, low grade precursor lesions and high-grade precursor lesions was analyzed by chi square analysis.

## Results

The clinicopathological features of the 420 BCa cases are summarized in Table 1. The mean age at diagnosis was 57.36 (SD = 11.96). The majority were invasive breast carcinomas, of no special type (374/420, 89%) and of Nottingham grade 3 (238/420, 56.7%). The majority was positive for oestrogen (323/420, 76.9%) and progesterone receptors (308/420, 73.3%). 140 tumours (33.3%) were positive for HER 2.

**Table 1:** Clinicopathological characteristics of the breast carcinoma study population

Clinicopathological characteristics	Frequency	Frequency (Valid percentage %)
**Age (Years)**		
Mean age (SD)	**57.36** (SD 11.96)	
**Age category**		
< 40 years	38	9%
**41 – 70 years**	**328**	**78%**
> 71 years	54	13%
**Histological subtypes of tumours**		
**Invasive carcinoma, NST**	**374**	**89%**
Lobular carcinoma	9	2.1%
Mucinous carcinoma	10	2.4%
Tubular carcinoma	1	0.2%
Invasive breast carcinoma with medullary pattern	5	1.2%
Invasive papillary carcinoma	1	0.2%
Invasive cribriform carcinoma	2	0.5%
Metaplastic carcinoma	1	0.2%
Micropapillary carcinoma	3	0.7%
Adenoid cystic carcinoma	2	0.5%
Pleomorphic lobular carcinoma	7	1.7%
Solid papillary carcinoma	3	0.7%
Tubulolobular carcinoma	1	0.2%
Mixed carcinoma (Mucinous + Ductal)	1	0.2%
**Nottingham grade**		
Grade 1	32	7.6%
Grade 2	150	35.7%
**Grade 3**	**238**	**56.7%**
**Hormone receptor status and HER 2 Overexpression**		
Oestrogen receptor status		
Positive	323	76.9%
Negative	97	23.1%
Progesterone receptor status		
Positive	308	73.3%
Negative	112	26.7%
HER 2 Overexpression		
0/1 + (Negative)	280	66.7%
3 + (Positive)	140	33.3%
**Surrogate molecular subtype**		
**Luminal like**	**323**	**76.7%**
HER 2 Positive (Non luminal)	44	10.5%
Triple negative	54	12.9%

**Fig. 2:**
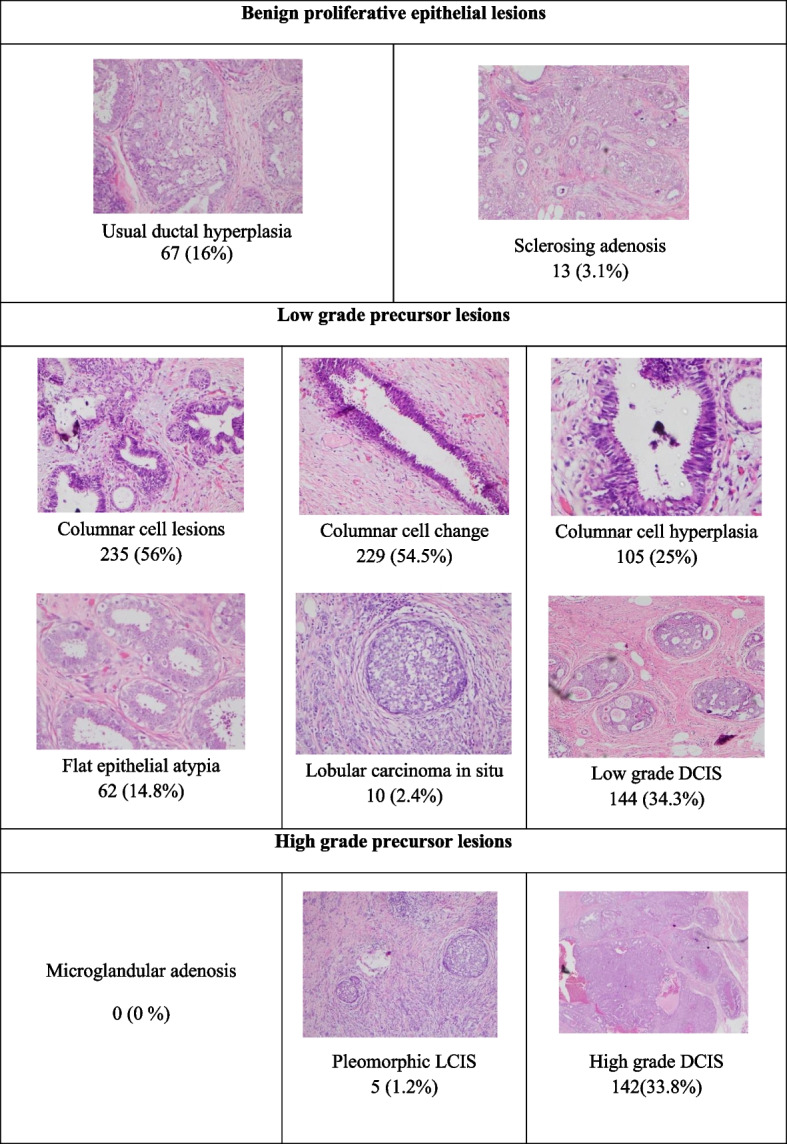
Frequencies of risk indicators and precursor lesions

All 420 cases were examined microscopically for benign proliferative breast lesions, low grade precursor lesions and high-grade precursor lesions and the findings are summarized Fig. 2.

### Tumour adjacent tissue in ER positive vs ER negative BCa

The associations of risk indicators and precursor lesions with ER status are summarized in Table 2. Most risk indicators, low grade precursor lesions and high grade precursor lesions were more commonly seen in ER positive BCa. ADH was not encountered in the study sample. Sclerosing adenosis and high-grade DCIS were more frequent in ER negative BCa. LCIS and pleomorphic LCIS were only seen in ER positive carcinomas.

**Table 2:** Association between proliferative epithelial changes in tumour adjacent tissue and ER status of the tumour

**Proliferative epithelial** **Changes in tumour** **Adjacent tissue**		**ER positive (*n* = 322)**	**ER negative(HER2/TN)(*n*** **= 98)**	***P* Value**
Usual ductal hyperplasia	Present	55 (17.08%)	12 (12.24%)	0.252
	Absent	267 (82.90%)	86 (87.75%)	
Sclerosing adenosis	Present	9 (2.79%)	4 (4.08%)	0.520
	Absent	313 (97.21%)	94 (95.92%)	
Columnar cell lesions	Present	188 (58.38%)	47 (47.95%)	0.069
	Absent	134 (41.62%)	51 (52.05%)	
Columnar cell change	Present	183 (56.83%)	46 (46.93%)	0.085
	Absent	139 (43.1%)	52 (53.07%)	
**Columnar cell hyperplasia**	**Present**	**88 (27.32%)**	**17 (17.34%)**	0.04 **
	Absent	234 (72.68%)	81 (82.66%)	
**Flat epithelial atypia**	**Present**	**54 (16.77%)**	**8 (8.16%)**	0.035 **
	Absent	268 (83.23%)	90 (91.84%)	
Lobular carcinoma insitu	Present	10 (3.10%)	0 (0%)	0.077
	Absent	312 (96.9%)	98 (100%)	
**Low grade DCIS**	**Present**	**133 (41.30%)**	**11 (11.22%)**	0.000 **
	Absent	189 (58.7%)	87 (88.78%)	
Pleomorphic LCIS	Present	5 (1.55%)	0 (0%)	0.215
	Absent	317 (98.45%)	98 (100%)	
**High grade DCIS**	**Present**	**99 (30.74%)**	**43 (43.87%)**	0.016 **
	Absent	223 (69.26%)	55 (56.13%)	

CCH (*p*-0.046), FEA (*p* = 0.035), LGDCIS (*p* < 0.001) showed a statistically significant association with ER positive tumours. HGDCIS showed a statistically significant association with ER negative tumours (*p* = 0.016).

## Tumour adjacent tissue in low grade (NG1/NG2) vs high grade (NG3) BCa

The associations of risk indicators and precursor lesions with tumour grade are summarized in Table 3.. CCC (*p* = 0.006), FEA(*p* = 0.048), LG DCIS (*p* < 0.001) showed a statistically significant association with low tumour grade (*p* = 0.005). A significant association was seen between high grade tumours and HG DCIS (*p* < 0.001).

**Table 3:** Association between proliferative epithelial changes in tumour adjacent tissue and Nottingham grade of the tumour

Proliferative epithelial changes in tumour adjacent tissue	Nottingham grade			***P*** Value
	Low grade		High grade	
	Grade 1 &2 (***n***=182)		Grade 3 (***n***=238)	
Usual ductal hyperplasia	Present	35 (19.23%)	32 (13.44%)	0.109
	Absent	147 (80.77%)	206 (86.56%)	
Sclerosing adenosis	Present	6 (3.29%)	7 (2.94%)	0.835
	Absent	176 (96.71%)	231 (97.06%)	
**Columnar cell lesions**	**Present**	**116 (63.73%)**	**119 (50%)**	0.005**
	Absent	66 (36.27%)	119 (50%)	
**Columnar cell change**	**Present**	**113 (62.08%)**	**116 (48.73%)**	0.006**
	Absent	69 (37.92%)	122 (51.27%)	
Columnar cell hyperplasia	Present	53 (29.12%)	52 (21.84%)	0.088
	Absent	129 (70.88%)	186 (78.16%)	
**Flat epithelial atypia**	**Present**	**34 (18.68%)**	**28 (11.76%)**	0.048**
	Absent	148 (81.32%)	210 (88.24%)	
Lobular carcinoma insitu	Present	7 (3.84%)	3 (1.26%)	0.085
	Absent	175 (96.16%)	235 (98.74%)	
**Low grade DCIS**	**Present**	**95 (52.19%)**	**49 (20.58%)**	0.000 **
	Absent	87 (47.81%)	189 (79.42%)	
Pleomorphic LCIS	Present	1 (0.54%)	4 (1.68%)	0.289
	Absent	181 (99.46%)	234 (98.32%)	
**High grade DCIS**	**Present**	**33 (18.13%)**	**109 (45.79%)**	0.000**
	Absent	149 (81.87%)	129 (54.21%)	

## Discussion

### Precursor lesions and risk indicators in tumour adjacent tissue

To date a detailed morphological study to evaluate the role of distinct proliferative epithelial changes of the breast for the development of breast carcinoma has not been undertaken in the Sri Lankan setting. In our study we evaluated 420 breast carcinomas for the presence of risk indicators (UDH, SA and radial scar), low grade precursor lesions (CCLs, ADH, LG DCIS, LCIS) and high grade precursor lesions (MGA, PLCIS, HG DCIS) in tumour adjacent tissue within 10 mm distance from tumour-host interface.

Two descriptive studies conducted in Asia analyzed the proliferative and non-proliferative histological changes in areas adjacent to breast cancer and demonstrated a similar spectrum of lesions to the current study. The most common lesions encountered were fibrocystic disease, epithelial hyperplasia, sclerosing adenosis, columnar cell lesions and DCIS [14, 15]. Both these studies demonstrated proliferative changes in 38–39% of the cases studied. However these studies did not compare the spectrum of changes according to ER status or tumour grade. CCC was the most commonly identified lesion in the current study followed by LG DCIS, HG DCIS, CCH and UDH.

### Association of precursor lesions/risk indicators with ER status and tumour grade

In the current study, we found that the frequency distribution of risk indicators and precursor lesions differ according to ER status and tumour grade, with most risk indicators and precursor lesions being more prevalent with ER positive tumours. Only sclerosing adenosis and HG DCIS were more frequent in ER negative tumours (Table 3.). Abdel Fatah et al. demonstrated more frequent distribution of CCLs (76% vs 10%), UDH (24% vs 13%) and lobular neoplasia (58% vs 8%) in ER positive low-grade tumours than ER negative high-grade tumours [16]. In his study HG DCIS with comedo (67%) and solid (36%) growth patterns were commonly encountered in high grade ER negative tumours.

### Columnar cell lesions – Columnar cell change, Colomnar cell hyperplasia and flat epithelial atypia

Many studies have analyzed the tumour background for the presence of columnar cell lesions in low grade breast tumours [6, 17–21]. Abdel-Fatah et al. studied 147 low grade breast tumours and found a significant association between low grade breast carcinomas and CCLs, CCH and FEA but not CCC [6]. Molecular genetic studies have confirmed that columnar cell lesions harbor recurrent chromosomal abnormalities such as loss of 16q and that these lesions should be considered as a clonal, neoplastic process rather than a hyperplastic process. These findings have led to the suggestion that columnar cell lesions represent the earliest stage of low grade ER positive breast neoplasia family [4, 9, 16, 21]. Simpson et al. demonstrated the close resemblance of histological, immunohistochemical and molecular genetic features of CCLs with ER positive DCIS and invasive carcinomas [21]. His study confirmed the role of CCLs as precursor lesions in ER positive breast neoplasia family [21].

In concordance with the above studies morphologically, most of these columnar cell lesions in our study were present in ER positive tumours. CCH (*p* = 0.046) and FEA (*p* = 0.035) showed statistically significant association with ER positive tumours in comparison to ER negative tumours. There was no significant association with CCC alone. Statistically significant associations were seen between low grade tumours and CCLs (*p* = 0.005), CCC (*p* = 0.006) and FEA (*p* = 0.048). FEA showed significant association with both NG and ER status of the breast cancers.The progression of CCC towards the FEA, morphologically demonstrates atypia and architectural complexity together with superadded allelic imbalances. Our findings support the fact that these acquired changes lead to nuclear atypia and a complex architecture and are needed to progress towards carcinoma.

### LCIS and pleomorphic LCIS

All ten cases of LCIS identified in the present study were from ER positive tumours, most of which were low grade. PLCIS was seen in five out of seven pleomorphic lobular carcinomas. All were ER positive tumours and most were high grade. None of the associations were statistically significant possibly due to the small number of cases.

### LG DCIS and HG DCIS

As pointed out, previous molecular studies have shown similar allelic imbalances between ER positive low grade invasive carcinomas and associated LG DCIS components. Buerger et al. studied 77 cases of invasive breast carcinomas with different morphological subtypes and found a significant genetic homology between low grade carcinoma and well differentiated DCIS and between grade 3 invasive breast carcinomas and poorly differentiated DCIS [22]. According to the molecular genetic pathway of breast carcinoma ER positive LG DCIS is associated with ER positive low grade breast carcinomas and ER negative HG DCIS is associated with ER negative high grade breast carcinomas [4, 6, 9, 16–18, 20, 22, 23].

In agreement with this hypothesis, we demonstrated a statistically significant association between ER positive breast carcinomas and LG DCIS (*p* < 0.001)and ER negative breast carcinomas and HG DCIS (*p* = 0.016). Similarly we showed a statistically significant association between low grade tumours and LG DCIS (*p* < 0.001) and high grade tumours and HG DCIS (*p* < 0.001).

The major proportion of our study population consisted of ER positive BCa, *n* = 322 (76.7%). Most of the tumours were of Nottingham grade 3, *n* = 238 (56.7%). There were 147 ER positive (45.6%), grade 3 tumours. Among these ER positive tumours, 97 (30.12%) were also positive for HER2. In this category of tumours with both ER and HER 2 positivity, 40 (41.23%) were associated with a high grade DCIS component. Twenty nine (29.8%) showed LG DCIS. According to the novel breast carcinogenesis pathway, a small proportion of high grade tumours progress from a preexisting low grade invasive component. These tumours have shown loss of 16q, as in low grade tumours. This luminal lineage progression can be associated with both high grade and low grade DCIS within the same tumours [4, 5, 7, 9]. In our study, 52 (36.11%) cases with LG DCIS showed an associated HG DCIS component. This raises the possibility that in our Sri Lankan study population there is a significant component of tumours with luminal lineage progression. However, we need more comprehensive studies backed by molecular analysis to validate this finding.

In summary, we found that CCH was associated with ER positive tumours and CCLs as a whole and CCC was associated with low-grade tumours. FEA and LGDCIS were associated with both ER positive and low-grade tumours (NG1/NG2) and HGDCIS was associated with ER negative breast carcinomas and high-grade tumours (NG 3). These morphological findings support the two molecular based breast cancer pathogenetic pathways of breast carcinoma also in the studied setting.

From a more practical view point pathologists need to be aware of the molecular pathway of breast carcinogenesis and should be able to identify the non-obligate precursor lesions confidently, especially CCLs including FEA. CCLs contain microcalcifications. These microcalcifications maybe detected radiologically leading to breast core needle biopsy of these lesions. The identification of these non-obligate precursors in a core biopsy will help the surgeon to decide on further treatment modalities when the biopsies are negative for an invasive carcinoma. Currently, surgical excision of the suspicious lesion depends on the presence of more worrying lesions such as ADH and DCIS. Pathologists should be aware of the possibility of having ADH and DCIS in the vicinity of FEA, which is explained by the molecular pathways. Additionally CCLs occur in a background with genetic instability. Multiple deep levels to rule out the possibility of ADH and DCIS is recommended whenever CCLs or FEA are encountered in core needle biopsies/excision specimens. Clinicians need to be informed of the significance of CCLs and FEA when the breast core needle biopsies are negative for ADH, DCIS or invasive carcinoma. Discussion at multidisciplinary team meeting is necessary and close clinical and radiological follow up may be warranted.

## Conclusion

CCLs especially FEA and low grade DCIS were the precursor lesions associated with ER positive tumours. High grade DCIS was the precursor lesion associated with ER negative tumours. These morphological findings support the two recently described molecular based breast cancer pathogenetic pathways of breast carcinoma in the studied setting as well.

## Data Availability

The datasets generated and analyzed during the current study are not publicly available, since specific consent for this was not obtained at the time of conducting the study, however they could be made available from the corresponding author on reasonable request.

## Abbreviations

BCa: Breast carcinoma
ER: Oestrogen receptor
NG: Nottingham grade
CCL: Columnar cell lesions
CCC: Columnar cell change
CCH: Columnar cell hyperplasia
FEA: Flat epithelial atypia
ALH: Atypical lobular hyperplasia
LCIS: Lobular carcinoma insitu
ADH: Atypical ductal hyperplasia
LGDCIS: Low grade ductal carcinoma insitu
MGA: Microglandular adenosis
PLCIS: Pleomorphic lobular carcinoma insitu
HGDCIS: High grade ductal carcinoma insitu
UDH: Usual ductal hyperplasia
SA: Sclerosing adenosis
HER2: Human epidermal growth factor receptor 2
PR: Progesterone receptor
ASCO-CAP: American society of clinical oncology/College of American Pathologists
WHO: World health organization
IHC: Immunohistochemistry
FISH: Flourescent insitu hybridization
TNBC: Tripple negative breast carcinoma
SPSS: Statistical packages for social sciences
NHSL: National Hospital of Sri Lanka
